# Associations between perceived barriers and benefits of using HIV pre-exposure prophylaxis and medication adherence among men who have sex with men in Western China

**DOI:** 10.1186/s12879-018-3497-7

**Published:** 2018-11-15

**Authors:** Ying Hu, Xiao-ni Zhong, Bin Peng, Yan Zhang, Hao Liang, Jiang-hong Dai, Ju-ying Zhang, Ai-long Huang

**Affiliations:** 1 Department of Health Statistics and Information Management, School of Public Health and Management, Chongqing Medical University, Chongqing, China; 2 Department of Epidemiology and Medical Statistics, School of Public Health, Guangxi Medical University, Nanning, China; 3 Department of Epidemiology and Health Statistics, School of Public Health, Xinjiang Medical University, Xinjiang, China; 4 Department of Epidemiology and Medical Statistics, School of Public Health, Sichuan University, Sichuan, China; 5 Key Laboratory of Molecular Biology on Infectious Diseases, Ministry of Education, Chongqing Medical University, Chongqing, China

**Keywords:** Pre-exposure prophylaxis, PrEP, MSM, Adherence, Barriers and benefits

## Abstract

**Background:**

To investigate the associations between the perceived barriers and benefits of using HIV pre-exposure prophylaxis medication, including worries about the side effects, disliking taking drugs, perceived burden of taking medication, positive expectations as to the efficacy of the drugs, favourable doctor-patient relationships, and medication adherence among men who have sex with men (MSM) to provide a target for improving medication adherence and reducing HIV infection among MSM.

**Methods:**

MSM were recruited in western China from April 2013 to October 2014, administered oral tenofovir (TDF) daily and followed up every 12 weeks for 2 years. At each follow-up, the medication rate was calculated based on the self-reported number of missed doses over 2 weeks, and then, the medication adherence was evaluated. The barriers and benefits perceived during medication were obtained by a self-administered questionnaire, and their effects on medication adherence were analysed by linear mixed models.

**Results:**

A total of 411 participants were enrolled in this study, and 1561 follow-up observation points were obtained. The average medication rate was 0.62 ± 0.37, and the medication rate increased with longer follow-up (*P* < 0.05). The medication rate was higher among MSM who were divorced (compared to those who were unmarried, *P* < 0.0001). MSM with more positive expectations as to the efficacy of the drugs showed higher rates of medication (P < 0.0001), while those who were more worried about side effects had a lower medication rate (*P* = 0.0208). In contrast, the dislike of taking the drugs and the burden perceived during medication had no effects on the actual medication rate of taking TDF (*P* > 0.05).

**Conclusion:**

How to obtain and maintain high medication adherence among MSM is the key to the PrEP intervention strategy for effective reduction of HIV infection. For MSM in China, we should deepen their understanding of the effectiveness and safety of PrEP and increase their confidence in PrEP, thereby improving their medication adherence.

**Trial registration:**

ChiCTR-TRC-13003849. Registered on 24/06/2013.

## Background

The report of the Joint United Nations Program on HIV/AIDS [1] points out that the number of HIV infections reached 1.8 million in 2016 and men who have sex with men (MSM) have a higher risk of infection. The Progress Report on AIDS Prevention and Treatment in China [2] shows that the HIV epidemic currently maintains a low- prevalence trend, with a higher-prevalence among MSM, especially in the western part of China [3]. In 2006, MSM accounted for 2.5% of new HIV infections in China, and the rate increased dramatically to 25.5% in 2017 [4, 5].

Pre-exposure prophylaxis (PrEP) has been considered the most promising biomedical HIV prevention strategy so far. The antiviral drug tenofovir (TDF) used in PrEP and combined therapy with TDF and emtricitabine (TDF-FTC) have been approved by the USFDA as preventive drugs for the MSM population [6], and in 2014, comprehensive PrEP guidelines were released [7]. Many international clinical studies have shown the effectiveness of PrEP in different groups, including MSM [8–10], women [11] and injection drug users [12, 13].

In addition, studies have also suggested that adherence to medication is crucial for the efficacy of PrEP [9, 11, 14–16]. The iPrEP study demonstrated that the once-daily oral PrEP drug resulted a 44% reduction in the incidence of HIV among MSM and transgender women, while pill use on 90% or more of days showed greater protection (73%), and it increased to 92% efficacy for participants with detectable drug levels [10]. However, a study conducted among African women unable to show the efficacy of oral PrEP due to their low adherence levels [17]. Therefore, an investigation into barriers and facilities associated with adherence to PrEP is required.

Factors from the perspectives of social, behavioural and psychological areas associated with PrEP adherence have been identified in clinical trials [9, 18–20]. However, in China, only one open-label clinical trial has been conducted among HIV-uninfected MSM in western China to assess the efficacy and safety of oral TDF in preventing HIV infection [21], but little is known about the barriers and facilities of PrEP adherence in China. Based on the fact that PrEP clinical trials have not been widely conducted in China, assessing participants’ feelings and attitudes during medication among the very first group of people involved in taking PrEP drugs is important and necessary. For instance, participants who perceive that there are side effects of drugs tend to show low adherence to medication, while sound doctor-patient relationships are a facilitator of adherence [22, 23].

Therefore, in this study, we aimed to investigate the changes in barriers and benefits that HIV-uninfected MSM perceive during the course of medication and examine the associations between perceived barriers and benefits of oral PrEP and actual medication adherence to provide a basis and some guidance for application and promotion of PrEP strategies among MSM in China.

## Methods

### Participants and study design

Participants were recruited from Chongqing, Sichuan, Xinjiang and Guangxi by non-probability sampling from April 2013 to October 2014, including via publishing information on gay websites and cooperating with non-governmental organizations. Inclusion criteria: (1) MSM aged 18–65 years; (2) self-reported negative or unknown HIV status; (3) had engaged in sex with male partners at least every two weeks; (4) had at least one or more same-sex partners one month before the trial; and (5) willing to take medicine under guidance and comply with follow-up arrangements. Exclusion criteria: (1) HBsAg- or anti-HBc-positive, (2) having a serious illness that investigators considered could possibly interfere with the interventions, follow-ups or assessments of the participants; (3) advanced cancer; (4) alcohol abuse within one year before entering the study; (5) receiving other drugs 3 months prior to screening; and (6) having a history of severe allergies.

A total of 575 MSM eligible were enrolled in the daily TDF group after the screening, and they were informed about the purpose and content of the study as well as the possible benefits and risks. All participants signed the informed consent and were given standard HIV prevention interventions, including HIV testing, counselling to reduce the risk of HIV infection, free condom distribution, and STI management.

### Procedures

Participants were asked to fill in the self-administered quantitative questionnaire for the baseline survey and at each follow-up conducted every 12 weeks, inquiring about their feelings or attitudes during medication, the number of missed pills, as well as the occurrence of adverse events within the last two weeks during the follow-up. In addition, HIV-1 serological detection, blood biochemical examination and haematological examination were performed. At each follow-up, a new round of drugs was supplied. In addition, every two weeks, our trained investigators contacted participants though QQ or telephone to learn about their medication use and provide counselling on adherence.

Participants stopped using the medication if one of the following occurred: confirmed HIV infection; serious adverse reactions; failure to participate in follow-up, planning to leave the research study, or other reasons; or medicine withdrawal for personal reasons.

This study follows the *Declaration of Helsinki* and *Good Clinical Practice* and has been approved and supervised by the Ethics Committee.

### Measures

#### Demographic characteristics

Participants reported their age, household registration, educational level, marital status and average monthly income in the baseline survey.

#### HIV-related characteristics

HIV-related characteristics included HIV counselling or testing previously, threat perception of HIV, and HIV knowledge, which was addressed by 13 questions concerning HIV infection and transmission (1 point for correct answer, 0 for incorrect answer; the total scores≥11 points was regarded as a high level of HIV knowledge). Additionally, the number of male sexual partners, the frequency of looking for sexual partners through the Internet and whether they had been diagnosed with sexually transmitted diseases (STDs) by doctors in the past six months were assessed in the baseline survey.

#### Perceived barriers during medication

The barriers included a dislike of taking the drugs, worries about the drug’s side effects, and the sense of burden perceived during medication. Participants were asked about the barriers perceived when taking TDF in the face-to-face follow-up surveys every 12 weeks. The barriers were rated as 1 point (not at all) to 5 points (always), and participants rated them according to their feelings during medication.

The dislike of taking drugs was made up of 3 items, including “I don’t like the taste”, “I don’t like the formulation” and “I think the drugs are hard to swallow”. Then, the average score of the three was taken. Standard Cronbach’s α coefficient varied between 0.72 and 0.79 over eight time points.

In addition, the sense of burden perceived during medication was evaluated by “I’m worried about that sexual partners know that I’m taking medicine”, and the worries about the drug side effects were accessed by “I’m worried about drug side effects” directly both at baseline and during follow-up.

#### Perceived benefits during medication

The benefits included positive expectations to medication and favourable doctor-patient relationships at baseline and during the follow-up surveys.

Participants were asked to provide an answer in response to “I think the drugs kept me safe from AIDS” to evaluate the positive expectations to medication, with the answer scored with 1 point (not at all) to 5 points (always).

The doctor-patient relationship consists of two items: “Doctors here are friendly to me, and they care about my health,” and “I trust the doctors here.” The Cronbach’s α coefficient was 0.71 at baseline and varied between 0.74 and 0.91 over the eight time points.

#### Definition of adherence

We used the medication rate in the last two weeks to assess the adherence of the participants. At each follow-up, the participants were asked to answer whether they missed some doses or not and to fill in the number of missed doses if they did. Medication rate = (14 − number of missed doses)/14, between 0 and 1.

### Statistics analysis

The enumeration data including demographic characteristics were expressed as frequency and rate, while measurement data were expressed as $\overline{X}\pm S$, median and range. Longitudinal data were analysed using a linear mixed model (LMM), reporting the β coefficient and 95% confidence intervals (95% CI). LMM was used to assess the change in medication rate over the follow-up and to evaluate which variables were associated with the trend of change. The medication rate in each follow-up was used as the outcome variable to fit the null model and the random intercept-slope model. After the addition of the level 1 explanatory variable (scores of the barriers and benefits perceived during medication) and the level 2 explanatory variable (demographic characteristics), the final model was fitted. The modelling of the linear mixed models was conducted using the Proc Mixed of SAS software. All statistical analyses were performed using SAS 9.4 software and SPSS 22.0 software. *P* < 0.05 was considered to have statistical significance.

## Results

Of 575 participants who completed the baseline survey in the TDF group, 141 who did not participate in at least one follow-up and 23 who did not report the number of missed doses at least once were excluded. Thus, a total of 411 MSM involved in at least one follow-up with a maximum of 8 and an average of 3.54 were included in the final analysis. A total of 1561 follow-up observation points were provided.

The demographical and HIV-related characteristics are shown in Table 1. The average age of the 411 MSM was 29.34 ± 7.85 years, and their age ranged from 18 to 61 years. Among the participants, 73.90% were urban residents, and 26.10% were rural residents; 90% graduated from high school and above, and 78.83% were unmarried. Nearly half (48.27%) had an average monthly income above 3000 yuan. The scores of HIV knowledge ranged from 0 to 13 with a median of 9, and only 26.76% had scores above 11. While 64.63 and 81.91% of participants declared that they had ever engaged in HIV counselling and testing, respectively, only 56.97% believed that AIDS is a great threat to themselves and their families. Only 40.63% had only one male sexual partner in the past six months, but only 9.34% had been diagnosed with an STD in the past six months.

**Table 1:** Demographic and HIV-related characteristic

Variables	Group	N	%
Age	18–25 years old	163	39.66
	26–35 years old	158	38.44
	older than 35 years old	90	21.90
Household registration^a^	Urban	303	73.90
	Rural	107	26.10
Education level^a^	Junior high or below	39	9.56
	Senior high	116	28.43
	Junior college	93	22.79
	College or above	160	39.22
Marital status	Unmarried	324	78.83
	Married	62	15.09
	Divorced/widowed	25	6.08
Monthly income(RMB)^a^	≤1000	63	15.59
	1001–3000	146	36.14
	3001–5000	138	34.16
	5001–10,000	47	11.63
	≥10,001	10	2.48
HIV knowlegde score	< 11	301	73.24
	≥11	110	26.76
HIV counseling^a^	Yes	265	64.63
	No	145	35.37
HIV testing^a^	Yes	335	81.91
	No	74	18.09
Perceived AIDS severity^a^	Moderate and low	135	32.93
	High	275	67.07
Perceived AIDS threat to themselves and family^a^	Moderate and low	176	43.03
	High	233	56.97
Number of male sexual partners in the past six months^a^	1	154	40.63
	2	89	23.48
	≥3	136	35.88
Looking for sexual partners though the Internet in the past six months^a^	Never	148	37.95
	Occasionally	153	39.23
	Always/Sometimes	89	22.82
Diagnosed with STD by doctors in the past six months^a^	Yes	38	9.34
	No	369	90.66

^a^Partially missing data (numbers might not add up to the total because of missing data)

**Fig. 1:**
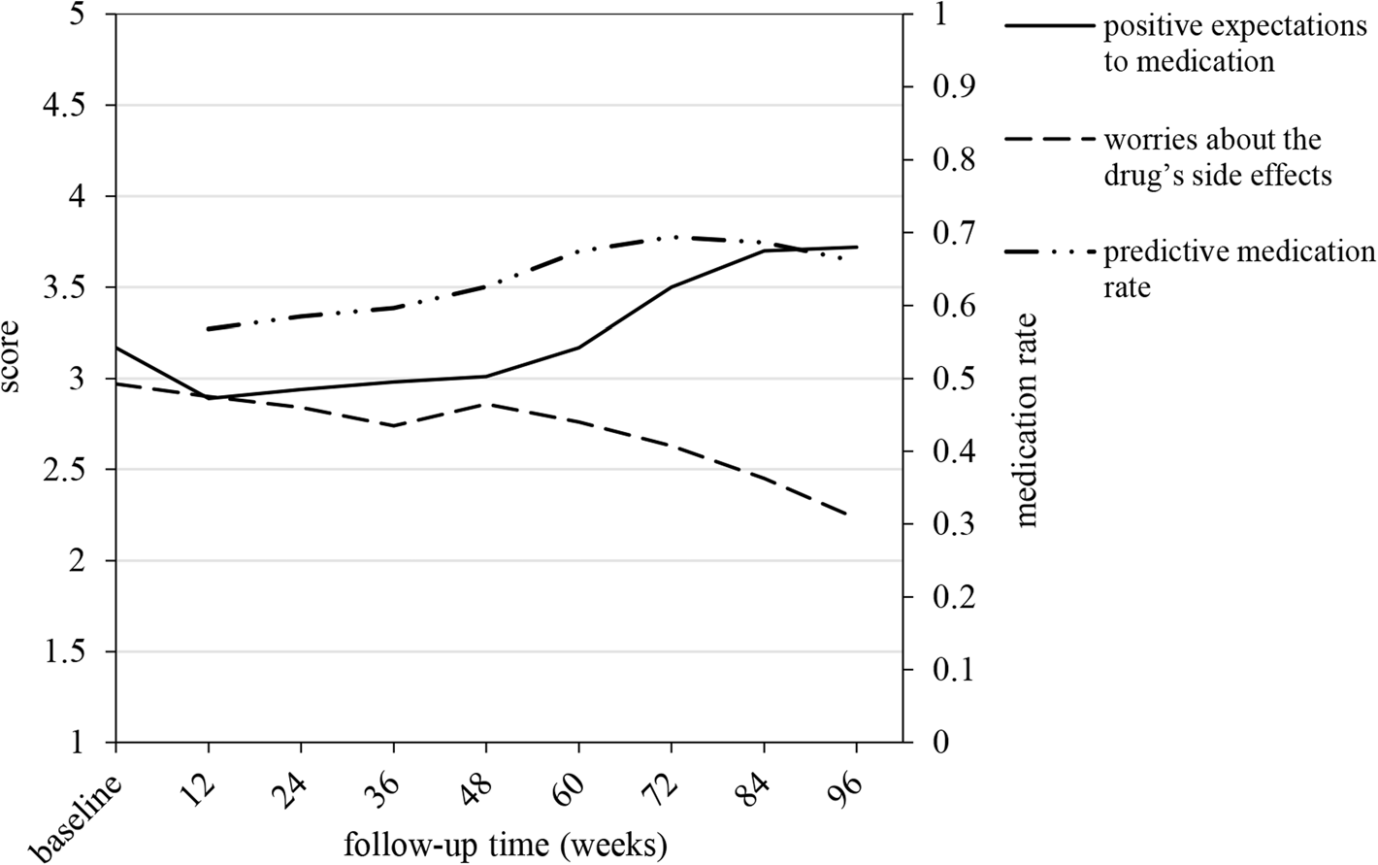
Graph of the changes in the scores of positive expevtations to medication and worries about the drug’s side effects and of predictive medication rate over the follow-up

The scores of the barriers and benefits participants perceived at baseline and during the follow-up are shown in Table 2. The changes in the scores of “worries about drug side effects” and “positive expectation about the efficacy of the drugs” over the follow-up are shown in Fig. 1. At baseline, when participants had not yet started to take PrEP, the score for “worries about drug side effects” was 2.97 ± 1.15, but it showed a decreasing trend during the follow-up. Participants had a relatively high expectation about the efficacy of the drugs at baseline (3.17 ± 1.36), and even though the score was 2.89 ± 1.41 at first follow-up, there was an increasing trend throughout the follow-up, which reached 3.72 ± 1.37 at the end. The scores of “disliked taking drugs” and “burden perceived during medication” fluctuated at a low and moderate level throughout the follow-ups, respectively. The score of “relationships between doctor and participants” was high both at baseline (4.20 ± 1.13) and throughout the entire follow-up.

**Table 2:** Overall medication rate and scores of the barriers and benefits perceived during medication (mean ± sd)

	Baseline	12 week	24 week	36 week	48 week	60 week	72 week	84 week	96 week
medication rate	Not applicable	0.57 ± 0.39	0.58 ± 0.37	0.59 ± 0.37	0.61 ± 0.36	0.70 ± 0.34	0.70 ± 0.33	0.69 ± 0.32	0.67 ± 0.39
dislike of taking drugs	Not applicable	1.69 ± 0.77	1.70 ± 0.84	1.65 ± 0.76	1.81 ± 0.87	1.75 ± 0.79	1.64 ± 0.75	1.70 ± 0.87	1.55 ± 0.77
worries about drugside effects	2.97 ± 1.15	2.90 ± 1.33	2.84 ± 1.42	2.74 ± 1.40	2.86 ± 1.45	2.76 ± 1.36	2.63 ± 1.37	2.45 ± 1.23	2.23 ± 1.07
burden perceived during medication	2.07 ± 1.23	2.23 ± 1.42	2.34 ± 1.50	2.32 ± 1.47	2.32 ± 1.53	2.54 ± 1.59	2.78 ± 1.66	2.21 ± 1.57	2.09 ± 1.41
positive expectations to medication	3.17 ± 1.36	2.89 ± 1.41	2.94 ± 1.42	2.98 ± 1.51	3.01 ± 1.54	3.17 ± 1.50	3.50 ± 1.51	3.70 ± 1.54	3.72 ± 1.37
favorable doctor patient relationship	4.20 ± 1.13	4.12 ± 1.16	3.92 ± 1.28	4.06 ± 1.24	4.02 ± 1.26	4.15 ± 1.17	4.30 ± 1.13	4.46 ± 1.03	4.52 ± 1.01

The average medication rate for the entire cohort was 0.62 ± 0.37, and its changes during the follow-ups are shown in Table 2. The results of the linear mixed model used to analyse the factors associated with medication adherence are shown in Table 3. The changes of predictive medication rate over the follow-up are shown in Fig. 1. The β coefficient for “time” (meaning follow-up times and coded as 1 to 8) was significant (β = 0.014; 95% CI = 0.005–0.024), indicating that the medication rate increased slightly during the follow-up. After adjusting for potential confounding variables, participants who were divorced showed a higher medication rate (compared with those who were unmarried, β = 0.179; 95% CI = 0.047–0.311). “Positive expectations towards medication”, as a time-varying value, was positively associated with the medication rate over time (β = 0.033; 95% CI = 0.020–0.047), indicating that those with higher scores on positive expectations as to the efficacy of the medicine over time reported better adherence to medication over time. In addition, the more worried the participant was about drug side effects during the follow-up, the lower the medication rate (β = − 0.019; 95% CI = − 0.035−− 0.003). However, the dislike of taking drugs, burden perceived during medication, and the relationship between the doctor and participant showed no correlation with the medication rate (*P* > 0.05).

**Table 3:** Linear mixed model for medication rate over time

	β	t value	*P*	95% CI
Variables				
Intercept	0.542	7.49	< 0.0001	0.400~ 0.683
Time	0.014	2.87	0.0046	0.005~ 0.024
Age	0.011	0.42	0.6717	−0.039~ 0.060
Household registration				
Rural	ref			
Urban	− 0.047	−1.20	0.2321	−0.123~ 0.030
Education level	0.018	1.07	0.2837	−0.015~ 0.052
Marital status				
Unmarried	ref			
Married	−0.017	−0.33	0.7429	−0.116~ 0.083
Divorced/widowed	0.179	2.65	0.0085	0.047~ 0.311
Monthly income(RMB)	−0.010	− 0.61	0.5440	− 0.044~ 0.023
Dislike of taking drugs	0.004	0.35	0.7267	−0.019 ~ 0.028
Positive expectations to medication	0.033	4.81	<.0001	0.020~ 0.047
Favorable doctor-patient relationship	−0.017	−1.94	0.0527	−0.033~ 0.001
Worries about drug side effects	−0.019	−2.31	0.0208	−0.035~ − 0.003
Burden perceived during medication	0.014	1.87	0.0621	−0.001~ 0.029

β: longitudinal linear regression coefficient for medication rate

## Discussion

This study is among the first to examine the potential factors related to PrEP adherence among MSM in a clinical trial in China, and its findings suggest that MSM’s positive expectations towards medication was a facilitator, while their worries about drug side effects was a barrier to PrEP adherence during the whole course of medication.

In the present study, a positive expectation towards medication refers to their trust in the efficacy of the drugs, which was evaluated only at a moderate level during the first follow-up but increased as the follow-up continued. The medication rate became higher along with an increase in their expectations during the follow-up, suggesting that it is still urgent to make efforts to publicize the effectiveness of PrEP drugs. Although many clinical trials of PrEP have been conducted worldwide and TDF was first approved by the FDA as a preventive drug for the MSM population in 2012, this is the first clinical research on PrEP conducted in China and according to the previous surveys on the willingness to use PrEP among MSM in China, only 22% of MSM (308/1402) reported that they had heard of PrEP [24], and a similar rate was observed among female sex workers (16.5%, 264/1611) [25] indicating that MSM in China only have a relatively low awareness of PrEP and that the majority of them have very little understanding of this new biomedical HIV prevention strategy. Therefore, providing more clinical evidence to them may contribute to improving their adherence to medication. It has always been said that the efficacy of drugs depends on patient adherence, but efficacy and adherence may complement each other since positive expectations as to their efficacy encourages people to be more likely to take the medicine on their own initiative.

In addition, the more worried patients are about side effects, the lower the medication rate, which is consistent with not only those studies examining factors associated with willingness to use PrEP [26] but also results found in clinical trials [27, 28]. Unlike therapeutic drugs, few people would take a preventative drug if it has serious side effects, which is in line with the findings of Mustanski et al. [29] that PrEP is more likely to be used as a preventative strategy for HIV if it has a low burden of side effects and satisfactory benefits are perceived. The worries about drug side effects showed a declining trend in the MSM during the follow-ups, which may be related to the low incidence of adverse events during medication. In our entire follow-up, the main adverse events were nausea, vomiting and diarrhoea, all with a low incidence, and there were no serious adverse events reported. Therefore, in addition to positive expectations of drug efficacy affecting the medication rate, their confidence in the safety of the drugs was also an important facilitator of their medication, which suggested that we should provide them correct information about this drug, inform them of possible side effects, and help them during the follow-ups, thereby reducing their worries about the efficacy and safety of the drugs and improve their adherence to medication.

There was no relationship between disliking taking drugs and the medication rate. In our study, the dislike of taking drugs referred to disliking the drug formulation, taste and pill size. The TDF used in this study were white tablets with 300 mg/tablet. During the follow-ups, the score of the dislike of taking drugs was lowest, which means the drug formulation, taste and size were acceptable by participants to some extent.

No association between the burden perceived during medication and the medication rate was observed. We hypothesized that taking medicine would give their partners a sense that they are in HIV at-risk status, which may impact their adherence. However, we did not find any such relationship and considered that this may be related to the dosing strategy, in which daily oral drugs had more flexibility when compared with dosing ‘on demand’, another dosing strategy recommended for MSM (a double dose of TDF/FTC 2–24 h before each sexual intercourse, followed by two single doses of TDF/FTC, 24 and 48 h after the first drug intake), and with this dosing strategy (dosed ‘on demand’), Mutua et al. [30] found that the burden perceived during medication reduced adherence.

However, the average medication rate during the entire follow-up was not very high. We believed that, other than the factors that we have examined in this study, there were some other barriers that existed and impacted the medication rate. In this study, we mainly focused on some attitudinal factors during medication and examined their relationships with adherence, which could provide some guidelines as to the implementation of clinical trials in China, and thus, it is the very first step in the promotion of PrEP in China. Nevertheless, medication is a long-term process, and long-term adherence to medication may be affected by many factors. For instance, patients may forgot to take their medicine, which has been reported in some studies [28, 31] and in our study; when we asked participants why they missed their doses, some reported that they forgot to take them sometimes. Thus, in the future, interventions should be developed to help remind people to take their medicine. In addition, supplying patients with additional correct information about the benefits and potential risks of the drugs, provided to both MSM and clinicians, their confidence in the drug efficacy could increase, and at the same time, their adherence could be improved.

### Limitations

There are some shortcomings in this study. First, we recruited the participants by non-probability sampling, which may lead to some bias and limit the generalizability of this study. In addition, even though we enrolled 575 MSM, 141 did not participate in at least one follow-up. However, when compared with those who were involved in all follow-ups, there were no significant differences in their attitudes (positive expectation about the efficacy of the drugs, worries about the drug side effects, the sense of burden perceived, doctor-participant relationship) in the baseline survey. The average number of follow-ups of the 411 participants was 3.5 times, and there was a certain loss to follow-up. Ensuring a high retention rate should be a priority in future clinical trials. In addition, we used the self-reported number of pills in the past two weeks to evaluate the medication rate. Self-report is the most widely used method to assess medication adherence; however, it may have some recall bias. [32]. Other approaches used in PrEP clinical trials in other countries such as pill counts, electronic monitoring device data, and blood drug level have their strengths and limitations, and therefore, a combination of two or more methods to evaluate adherence could be used in the next clinical trial [33].

## Conclusion

How to obtain and maintain high medication adherence is the key in PrEP to achieve an effective reduction in HIV infection. The results of our study showed that the overall medication rate in the daily medication group is not high. Worries about the efficacy and safety of PrEP were factors influencing the medication rate. For MSM in China, we should work to increase their understanding of the effectiveness and safety of PrEP and increase their confidence in PrEP, thereby improving their medication adherence.

## Abbreviations

MSM: Men Who Have Sex With Men Men Who Have Sex With Men
PrEP: Pre-exposure Prophylaxis
TDF: Tenofovir
TDF-FTC: Tenofovir and Emtricitabine
USFDA: US Food and Drug Administration

## References

[1] UNAIDS (2017). UNAIDS DATA 2017.

[2] 2015 China AIDS Response Progress Report. Available from: http://www.unaids.org/sites/default/files/country/documents/CHN_narrative_report_2015.pdf. Accessed 9 Apr 2018.

[3] Zhang L (2013). HIV prevalence in China: integration of surveillance data and a systematic review. Lancet Infect Dis.

[4] Qin Q (2017). Spatial analysis of the human immunodeficiency virus epidemic among men who have sex with men in China, 2006-2015. Clin Infect Dis.

[5] NCAIDS N (2017). Update on the AIDS/STD epidemic in China in December. Chinese J AIDS&STD.

[6] Holmes David (2012). FDA paves the way for pre-exposure HIV prophylaxis. The Lancet.

[7] Smith DK (2014). Preexposure prophylaxis for the prevention of HIV infection in the United States—2014: a clinical practice guideline. Korean J Ophthalmol.

[8] Thigpen MC (2012). Antiretroviral preexposure prophylaxis for heterosexual HIV transmission in Botswana. N Engl J Med.

[9] Grant RM (2014). Uptake of pre-exposure prophylaxis, sexual practices, and HIV incidence in men and transgender women who have sex with men: a cohort study. Lancet Infect Dis.

[10] Grant RM (2010). Preexposure chemoprophylaxis for HIV prevention in men who have sex with men. N Engl J Med.

[11] Karim QA (2010). Effectiveness and safety of tenofovir gel, an antiretroviral microbicide, for the prevention of HIV infection in women. Science.

[12] Choopanya K (2013). Antiretroviral prophylaxis for HIV infection in injecting drug users in Bangkok, Thailand (the Bangkok Tenofovir study): a randomised, double-blind, placebo-controlled phase 3 trial. Lancet.

[13] Alistar SS, Owens DK, Brandeau ML (2014). Effectiveness and cost effectiveness of oral pre-exposure prophylaxis in a portfolio of prevention programs for injection drug users in mixed HIV epidemics. PLoS One.

[14] Haberer JE (2013). Adherence to antiretroviral prophylaxis for HIV prevention: a substudy cohort within a clinical trial of Serodiscordant couples in East Africa. PLoS Med.

[15] Marrazzo JM (2015). Tenofovir-based Preexposure prophylaxis for HIV infection among African women. N Engl J Med.

[16] Muchomba FM (2012). State of the science of adherence in pre-exposure prophylaxis and microbicide trials. J Acquir Immune Defic Syndr.

[17] Van DL (2012). Preexposure prophylaxis for HIV infection among African women. N Engl J Med.

[18] Mehrotra ML (2016). The effect of depressive symptoms on adherence to daily Oral PrEP in men who have sex with men and transgender women: a marginal structural model analysis of the iPrEx OLE study. Aids & Behavior.

[19] Defechereux PA (2016). Depression and Oral FTC/TDF pre-exposure prophylaxis (PrEP) among men and transgender women who have sex with men (MSM/TGW). Aids & Behavior.

[20] Minnis AM (2016). Pre-exposure prophylaxis adherence measured by plasma drug level in MTN-001: comparison between vaginal gel and oral tablets in two geographic regions. Aids & Behavior.

[21] Zeng X. Tenofovir-based oral PrEP prevents HIV infection among men who have sex with men in western China:a multicenter, randomized, controlled clinical trial: Chongqing Medical University; 2013 (in Chinese).

[22] O’Brien MK, Petrie K, Raeburn J (1992). Adherence to medication regimens: updating a complex medical issue. Med Care Review.

[23] Bakken S (2000). Relationships between perception of engagement with health care provider and demographic. AIDS Patient Care STDs.

[24] Zhang Y (2013). Attitudes toward HIV pre-exposure prophylaxis among men who have sex with men in western China. Aids Patient Care & Stds.

[25] Peng B (2012). Willingness to use pre-exposure prophylaxis for HIV prevention among female sex workers: a cross-sectional study in China. Hiv/aids.

[26] Holloway IW (2017). Facilitators and barriers to pre-exposure prophylaxis willingness among young men who have sex with men who use geosocial networking applications in California. Aids Patient Care & Stds.

[27] Gengiah TN (2014). Adherence challenges with drugs for pre-exposure prophylaxis to prevent HIV infection. Int J Clin Pharm.

[28] Kebaabetswe PM (2015). Factors associated with adherence and concordance between measurement strategies in an HIV daily Oral Tenofovir/Emtricitibine as pre-exposure prophylaxis (Prep) clinical trial, Botswana, 2007-2010. Aids & Behavior.

[29] Mustanski B (2013). Perceived likelihood of using HIV pre-exposure prophylaxis medications among young men who have sex with men. Aids Behavior.

[30] Mutua G (2012). Safety and adherence to intermittent pre-exposure prophylaxis (PrEP) for HIV-1 in African men who have sex with men and female sex workers. PLoS One.

[31] Skoler-Karpoff S (2008). Efficacy of Carraguard for prevention of HIV infection in women in South Africa: a randomised, double-blind, placebo-controlled trial. Lancet.

[32] Williams AB (2013). A proposal for quality standards for measuring medication adherence in research. Aids & Behavior.

[33] Abaasa A (2017). Utility of different adherence measures for PrEP: patterns and incremental value. Aids & Behavior.

